# Analyses of a Panel of Transcripts Identified From a Small Sample Size and Construction of RNA Networks in Hepatocellular Carcinoma

**DOI:** 10.3389/fgene.2019.00431

**Published:** 2019-05-08

**Authors:** Zhiyong Sheng, Xiaolin Wang, Geliang Xu, Ge Shan, Liang Chen

**Affiliations:** ^1^Anhui Province Key Laboratory of Hepatopancreatobiliary Surgery, First Affiliated Hospital of University of Science and Technology of China, Hefei, China; ^2^Hefei National Laboratory for Physical Sciences at Microscale, The Chinese Academy of Sciences (CAS) Key Laboratory of Innate Immunity and Chronic Disease, School of Life Sciences, University of Science and Technology of China, Hefei, China; ^3^Chinese Academy of Sciences (CAS) Centre for Excellence in Molecular Cell Science, Shanghai Institutes for Biological Sciences, CAS, Shanghai, China

**Keywords:** hepatocellular carcinoma, lncRNA, miRNA, circRNA, network

## Abstract

Hepatocellular carcinoma (HCC) is one of the most common cancers in the world. Dysregulation of mRNAs and non-coding RNAs (ncRNAs) plays critical roles in the progression of HCC. Here, we investigated HCC samples by RNA-seq and identified a series of dysregulated RNAs in HCC. Various bioinformatics analyses established long non-coding RNA (lncRNA)-mRNA co-expression and competing endogenous RNA (ceRNA) networks in circRNA-miRNA-mRNA axis, indicating the potential *cis* and/or *trans* regulatory roles of lncRNAs and circRNAs. Moreover, GO pathway analysis showed that these identified RNAs were associated with many biological processes that were related to tumorigenesis and tumor progression. In conclusion, we systematically established functional networks of lncRNA-mRNA, circRNA-miRNA-mRNA to further unveil the potential interactions and biological processes in HCC. These results provide further insights into gene expression network of HCC and may assist future diagnosis of HCC.

## Introduction

Hepatocellular carcinoma (HCC) is one of the most frequent malignancies worldwide, and the highest incidence rates in the world are reported in Asia and Africa, with China alone accounting for half of the cases in Asia and Africa (McGlynn and London, 2011). Clinical investigation shows that HCC is the sixth most common cancer and third main cause of cancer mortality in the world (Forner et al., 2018). The overall 5 years survival rate is less than 20% due to lack of early and timely detection and treatment (Lee et al., 2012). So far, the available treatment approaches for HCC mainly include resection, liver transplantation, image-guided tumor ablation, and systemic therapy. The 5 years survival rates of 60–70% can be achieved in early-stage HCC patients (Llovet and Bruix, 2003; Llovet et al., 2003). However, systematic therapies cannot improve the survival rates of the patients with advanced stage of HCC due to limited specific and effective biomarkers or targets for clinical treatments (Llovet and Bruix, 2003; Llovet et al., 2003; Marquardt et al., 2012).

Dysregulated gene expression is a common theme underneath human disease (Adams and Cory, 2007; Aguirre-Ghiso, 2007; Amaravadi and Thompson, 2007; Azam et al., 2010; Hanahan and Weinberg, 2011). The resulted abnormal mRNAs and non-coding RNAs (ncRNAs) play roles in the occurrence and progress of diseases such as cancers (Prensner et al., 2011; Kung et al., 2013; Iyer et al., 2015; Rusk, 2015; Klingenberg et al., 2017). Many mRNAs are encoded by tumor genes or tumor suppressor genes, and ncRNAs such as microRNAs (miRNAs), long ncRNAs (lncRNAs), and circular RNAs (circRNAs) are also known to be associated with tumorigenesis and tumor progression (Panzitt et al., 2007; Wang et al., 2010; Wei et al., 2012; Zhang et al., 2012; Li G. et al., 2015; Palazzo and Lee, 2015; Zhou et al., 2016; Cui et al., 2017; Shen et al., 2018; Zhang and Wu, 2018).

In HCC, tumor genes such as procollagen-lysine, 2-oxoglutarate 5-dioxygenase 3 (PLOD3), and splicing factor 3b subunit 4 (SF3B4) are overexpressed in HCC tumor tissues compared with adjacent tissues, and knocking down their expression levels can effective suppress tumor invasion (Shen et al., 2018). Conversely, tumor suppressor genes such as Alcohol dehydrogenase 4 (ADH4) is significantly downregulated in tumor tissues, and patients with lower ADH4 expression levels have worse prognosis and lower overall survival rate (Wei et al., 2012).

In HCC, many miRNAs are found to play oncogenic or tumor suppressive roles (Zhang et al., 2012; Li G. et al., 2015; Zhou et al., 2016). For example, miR-421 can promote proliferation and migration in HCC cell by downregulating farnesoid X receptor (FXR), which is usually highly expressed in normal liver (Zhang et al., 2012). On the other hand, miR-708 is markedly downregulated in HCC tumor tissues compared with adjacent tissues. Low miR-708 level is related to tumor node metastasis (TNM) in advanced stage patients, and over expression of miR-708 can suppress the invasion and migration of in HCC cell lines *in vitro* (Li G. et al., 2015).

LncRNAs are also correlated to HCC (Chen et al., 2018). The lncRNA HULC is upregulated in HCC with high specificity (Panzitt et al., 2007; Wang et al., 2010). It can be detected both in tumor tissues and blood of HCC patients. Aberrantly elevated HULC promotes HCC invasion and progression by increasing expression of the oncogene HMGA2. The lncRNA PVT1 enhances the tumorigenesis and metastasis of HCC by acting as a competing endogenous RNA (ceRNA) for miR-186-5p, and the knockdown of PVT1 can effectively impede HCC invasion (Karreth et al., 2015; Cui et al., 2017).

CircRNAs are a large class of transcripts in multicellular organisms with emerging importance (Chen and Shan, 2015; Chen et al., 2015; Haque and Harries, 2017). One of functional mechanisms of circRNAs is acting as ceRNA (Hansen et al., 2013; Memczak et al., 2013). For example, the circRNA CDR1as functions as sponge of miR-7 in neuronal tissues (Memczak et al., 2013; Piwecka et al., 2017). Alterations in circRNA expression are found to be related to aberrant physiology and human diseases (Hansen et al., 2013; Memczak et al., 2013; Chen and Shan, 2015; Chen et al., 2015; Holdt et al., 2016; Piwecka et al., 2017).

In this study, we have identified differentially expressed mRNAs, miRNAs, lncRNAs, and circRNAs in fresh HCC tissues through high-throughput RNA sequencing. Functional networks of lncRNA-mRNA and circRNA-miRNA-mRNA have been established to provide new insights for biomarkers and treatments in HCC.

## Materials and Methods

### Clinical Samples

All fresh HCC patient tumor samples and adjacent tissues were collected from The First Affiliated Hospital of University of Science and Technology of China, which was approved by the Human Research Ethics Committee of University of Science and Technology of China (USTCEC201700007). Written informed consent was obtained from each patient for this study. All samples were rinsed with DEPC water and then kept in RNAhold (TransGen) within 30 min after removing from the operation. HCC patient tumor sample and adjacent tissue pairs were collected from 21 patients (12 males and 9 females with advanced stage HCC, all of them were HBsAg positive, and did not have anti-tumor therapy before surgery).

### Total RNA Extraction

The clinical samples were cut into small pieces and homogenized in TRizol reagent (Life Technologies) by homogenizer. Total RNA was extracted by using TRizol reagent according to the manufacturer’s instructions.

### Transcriptome Data Analysis

Total RNAs from four pairs of HCC patient tumor tissues were extracted for high-throughput sequencing. Whole transcriptome libraries were constructed by the TruSeq Ribo Profile Library Prep Kit (Illumina, United States), according to the manufacturer’s instructions. In brief, 10 μg total RNA was depleted rRNA with an Illumina Ribo-Zero Gold kit and purified for end repair and 5′-adaptor ligation. Then, reverse transcription was performed with random primers containing 3′ adaptor sequences and randomized hexamers. Finally, the cDNAs were purified and amplified with thermo cycler. The PCR products of 300–500 bp were purified, quantified and stored at -80°C before sequencing. The libraries were subjected to 151 nt paired-end sequencing with an Illumina Nextseq 500 system (Novogene, China). Each library was generated a depth of 50–100 million read pairs and then adapters were removed with cutadapt to obtain clean reads. For mRNA and lncRNA analyses, the expression levels were calculated using TopHat2 and Cufflinks followed by the annotation references of Refseq and Ensemble transcript databases with the genome release Homo sapiens, hg19. The differentially expressed mRNA and lncRNA were determined by DEseq2 with the corresponding cutoff (*P* < 0.001, RPKM ≥ 10, | log2(fold change)|≥ 1 for mRNA and *P* < 0.05, RPKM ≥ 1, | log2(fold change)|≥ 1 for lncRNA). For circular RNA (circRNA) prediction, we identified the candidates with find_circ (Memczak et al., 2013) and the junction reads were calculated as Transcripts Per Kilobase Million (TPM). A criterion of *P* ≤ 0.05, TPM ≥ 0.1 and | log2(fold change)|≥ 1 among four pairs of samples was used to identify differentially expressed circRNAs. RNA-seq data were deposited in NCBI with the GEO accession code GSE128274.

### Small RNA Data Analysis

For small RNA (sRNA) sequencing, eight sRNA libraries were generated with TruSeq small RNA (Illumina, United States) according to the manufacturer’s instructions. Then the prepared libraries were sequenced with an Illumina Nextseq 500 system (Novogene, China). After filtering out the reads shorter than 15 nt, the remaining reads were mapped to the human genome (hg19) and the miRNA database in miRBase with bowtie (-v 1). The differentially expressed miRNAs were determined by DEseq2 with the cutoff of *P* < 0.05, TPM ≥ 1, | log2(fold change)|≥ 1.

### Construction of Co-expression and CeRNA Network

For the co-expression network of significantly dysregulated lncRNAs and mRNAs, Pearson’s correlations were calculated the co-expression analysis according to the expression levels in eight samples. A criterion of the coefficient parameter R-squared more than 0.99 was used for the remaining RNAs to further construct the network. For the competing endogenous RNAs (ceRNA) network of significantly dysregulated circRNAs and mRNAs, the miRNA/mRNA and miRNA/circRNA interaction were predicted with TargetScanHuman7.2. The above networks were both performed with Cytoscape^1^.

### GO Analysis

The significantly dysregulated mRNAs in co-expression and ceRNA network were both analyzed using GOrilla web-server with default parameters (Eden et al., 2009).

### Statistical Analysis

In all experiments, Student’s *t*-tests were used to calculate *P*-values, as indicated in the figure legends. The values reported in the graphs represent averages of actual number of independent experiments, with error bars showing SD. After analysis of variance with *F*-tests, the statistical significance and *P*-values were evaluated with Student’s *t*-tests.

### Reverse Transcription and Real-Time Quantitative PCR

cDNA was prepared using GoScript Reverse Transcription System (Promega) according to the manufacturer’s protocol. Notably, for the first strand cDNA synthesis of miRNA, stem-loop method was used (Kramer, 2011). Quantitative real-time PCR was performed with GoTaq SYBR Green qPCR Master Mix (Promega) on a PikoReal 96 real-time PCR system followed by 40 amplification cycles (Thermo Fisher Scientific) according to standard procedures. Actually, all amplification curves already reached stationary stage before 35 amplification cycles, and the readings of Ct value were obtained at the exponential stage. Relative RNA expression was normalized to ACTB expression level. All primers are shown in Table 1.

**Table 1:** Primers for RT-qPCR validation.

Name	Primer sequence 5′–3′	Product size (bp)
C12orf75	F: GCAGGAGCAGCCAAAGATGT R: CTGGACACCATATTGACAGC	113
ZIC5	F: AGCCCTTCAAGGCCAAATAC R: GCTTGTGGATCTTGAGGTTC	129
PZP	F: TCAAGCTAGAAGCTGGCATC R: AAGCACAAATTCCTCCACGG	137
FAM65C	F: TGACATCGCCGACTTCTTCA R: TACAAGGAGCCCTTCCTGCT	177
C1QL1	F: CACCTACCATGTCCTCATGC R: GCGTAGTCGTAGTTCTGGTC	120
TMEM74	F: GGAGCTCGCCATCAGCAAAA R: TTCCCTGAAGACGTGGCTTC	142
GNAZ	F: CATCGCCGCAGCTGACTATA R: CCACGTCCACCATCTTGAAG	132
LNC-TOB2P1	F: GTGACTACAGGAAGGTTAGC R: GAGAAGAGGCTGCATGAGAG	161
LOC100499489	F: TAGAGATGAGACGATGCAGG R: CAGCTCCACCCACGTGGTTT	110
LINC01093	F: AAGAGACTGGAGCCCTGAGT R: GCTGTAACCCCAAAAACTCC	92
LOC100130899	F: GAGGTTTGCTGCAAATCAGG R; ACATACTCACGGCTGTGCCT	101
LOC200772	F: GAGGTTTGCTGCAAATCAGG R; ACATACTCACGGCTGTGCCT	135
LNC-FENDRR	F: AGGACCATTCAGCTCTAGAG R: ATGGGGTGAGCAAAGCGCAT	141
LNC-NBPF22P	F: CCATGTCACCACCAAGAACA R: CCATGTCACCACCATCAATG	152
LNC-NMRAL2p	F: TGCAACACCGAAGGATTTCC R: CAAGTTCCGAGGCTTCTCAT	148
ACTB	F: CCAACACAGTGCTGTCTGG R: GAGTACTTGCGCTCAGGAG	130
PLOD3	F: GTTTGTGGATAGCTACGACG R: CCACTCTGGACGAACTTCTT	72
SF3B4	F: GGACCCTGAGATTGATGAGA R: GTCAGGGTCCCGCATAATTT	91
SNHG7	F: TGCTCACTGGAGATGACACG R: CACTGGAAGTCCATCACAGG	92
HULC	F: AGGAAGAGTCGTCACGAGAA R: TATTCCGGCCTTTACTTCAG	124
ADH4	F: ATCAACAATGCCAAGGTCAC R: CCTATGATTCTGGAAGCTCC	113
COLEC10	F: GGCAATATTGGCAAGACTGG R: TCTCCAGGTATTCCAAGCAA	77
circAKR1B10	F: CCTTGTGAGGAAAGCCTTTG R: CAGCACACTTAGAGGAAGCT	199
circAKR1C3	F: AGGATTGGCCAAGTCCATCG R: CCATCGTTTGTCTCGTTGAG	211
circHMGCS1	F: CTCGGATGTTGCTGAATGAC R: CGGTCTAATGCACTGAGGTA	197
circC3P1	F: GGTTGCAAGAACAGGAATCG R: TCACATGCTGTTGTCTGGAG	137
miR-10b-3p	SL: GTCGTATCCAGTGCAGGGTCCGAGGTATTCGCACTGGATACGACATTCCC	
	F: CACGCAACAGATTCGATT R: CCAGTGCAGGGTCCGAGGTA	
miR-421	SL GTCGTATCCAGTGCAGGGTCCGAGGT	
	ATTCGCACTGGATACGACGCGCCC	
	F: CACGCAATCAACAGACATTA R: CCAGTGCAGGGTCCGAGGTA	
miR-761	SL GTCGTATCCAGTGCAGGGTCCGAGGTATTCGCACTGGATACGACTGTGTC F: CACGCAGCAGCAGGGTGAAA R: CCAGTGCAGGGTCCGAGGTA	
miR-200a-3p	SL: GTCGTATCCAGTGCAGGGTCCGAGGTATTCGCACTGGATACGACACATCG	
	F: CACGCATAACACTGTCTGGT R: CCAGTGCAGGGTCCGAGGTA	
miR-200b-3p	SL: GTCGTATCCAGTGCAGGGTCCGAGGTATTCGCACTGGATACGACTCATCA	
	F: CACGCATAATACTGCCTGGT R: CCAGTGCAGGGTCCGAGGTA	
miR-139-5p	SL: GTCGTATCCAGTGCAGGGTCCGAGGTATTCGCACTGGATACGACACTGGA	
	F: CACGCATCTACAGTGCACGT R: CCAGTGCAGGGTCCGAGGTA	

*SL, Stem loop RT primer.*

## Results

### Identification of Differentially Expressed RNAs in HCC Samples

**FIGURE 1:**
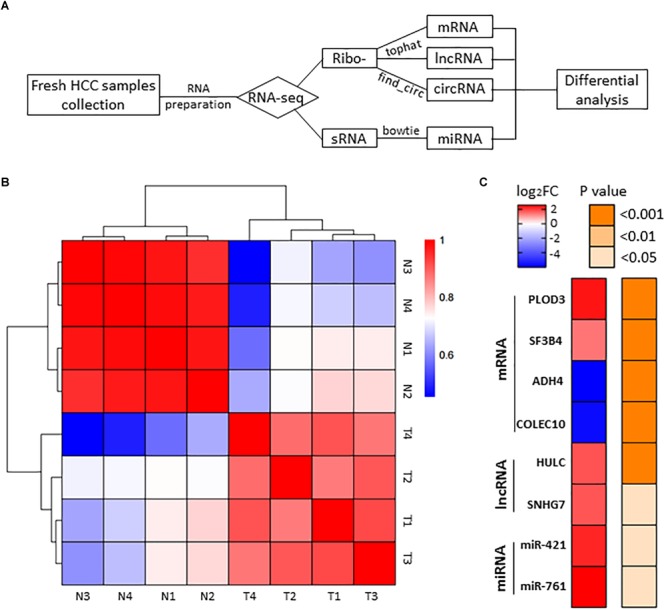
RNA-seq analysis of HCC samples. **(A)** The workflow of RNA-seq. **(B)** Heatmap of correlation between each sample by Pearson’s correlation coefficient analyses. **(C)** Eight RNA samples as positive controls are validated in the RNA-seq. Red, upregulated; blue, downregulated. Fold change ≥2.0, *P* < 0.05.

To identify HCC-related RNAs, we used four HCC patients’ fresh tumor tissues and paired adjacent non-tumor tissues for RNA sequencing. Differential expression of mRNAs, lncRNAs, circRNAs, and miRNAs were then analyzed (Figure 1A). Pearson’s correlation coefficient analysis showed that tumor tissues were positively correlated with each other, and control tissues also showed strong positive correlation (Figure 1B). It seems evident that the 4 tumor samples differ more from normal samples than among themselves, maybe due to the tumor heterogeneity. We noticed that previously reported transcripts with aberrant levels in HCC such as mRNAs (PLOD3, SF3B4, ADH4, and COLEC10), lncRNAs (HULC and SNHG7), and miRNAs (miR-421 and miR-761) were also identified in our RNA-seq (Figure 1C; Panzitt et al., 2007; Wang et al., 2010; Wei et al., 2012; Zhang et al., 2012; Li G. et al., 2015; Zhou et al., 2016; Shen et al., 2018; Zhang and Wu, 2018).

**FIGURE 2:**
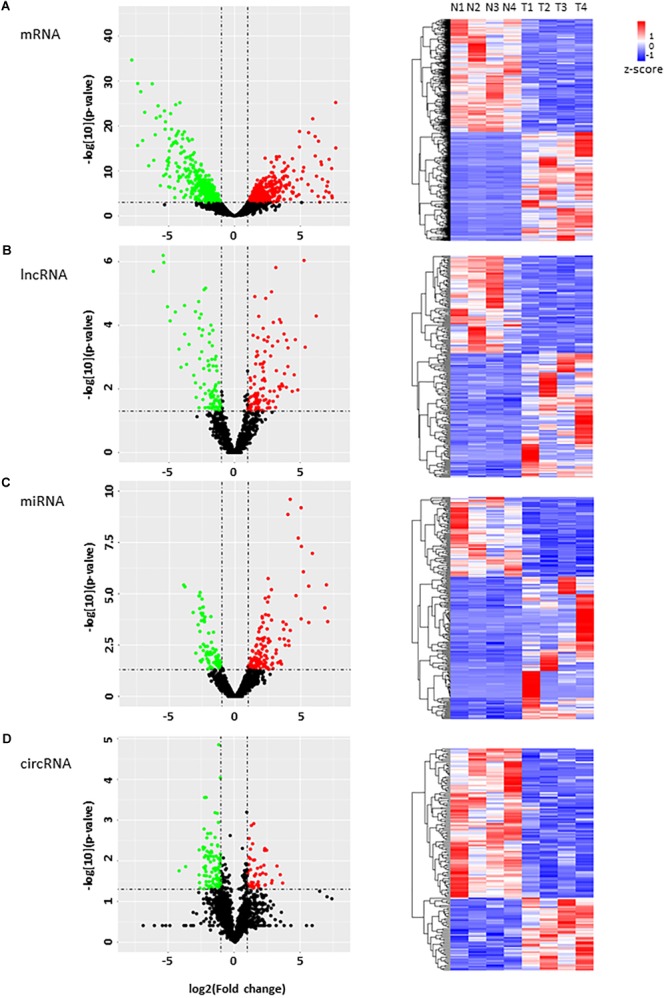
Analysis of the RNA expression profile in HCC. **(A–D)** volcano plots (left) display differentially expressed transcripts, and the hierarchical cluster analyses on the right of each panel display differential expression of RNAs in four HCC tumor tissues and their controls. **(A)** mRNAs, **(B)** lncRNAs, **(C)** miRNAs, and **(D)** circRNA. Green and red dots represent downregulated and upregulated RNAs respectively in tumor tissues compared with adjacent tissues. Black dots indicate no significant difference.

Next, we performed volcano plots for mRNAs, lncRNAs, miRNAs and circRNAs in HCC paired tissues (fold change ≥ 2.0, *P* < 0.05) (Figure 2A–D). 919 differentially expressed mRNAs, 207 lncRNAs, 216 miRNAs, and 152 circRNAs were identified (Figure 2A–D). Among them, 452 mRNAs (49.18%), 116 lncRNAs (50.04%), 138 miRNAs (63.89%), and 50 circRNAs (32.89%) were upregulated, while 467 mRNAs (50.82%), 91 lncRNAs (43.96%), 78 miRNAs (36.11%), and 102 circRNAs (67.11%) were downregulated (Figure 2A–D). We also conducted a hierarchical cluster analysis to display the differential expression of four types of RNAs across eight samples (Figure 2A–D). Tumor and adjacent non-tumor samples respectively were classified into different branch (Figure 2A–D).

### RT-qPCR Verification of the Dysregulated RNAs in Clinical Samples

**FIGURE 3:**
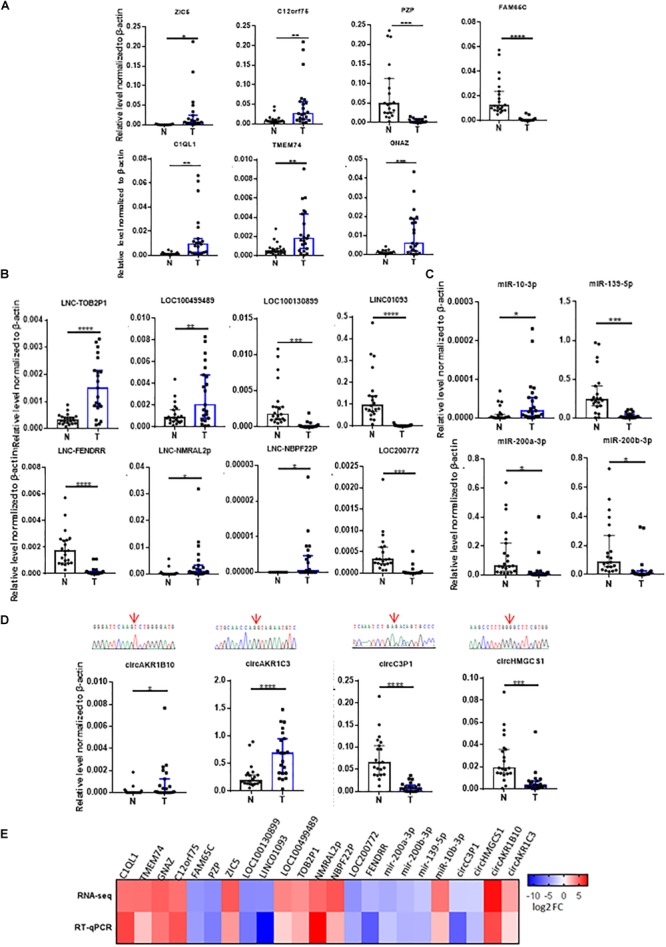
Validation of RNAs identified from RNA-seq in 21 HCC tumor tissues by RT-qPCR. The validation results of **(A)** 7 dysregulated mRNAs, **(B)** 8 dysregulated lncRNAs, **(C)** 4 dysregulated microRNAs, and **(D)** 4 dysregulated circRNAs, red arrows above indicate junctions of circRNAs from the sequencing results. **(E)** The heatmap for the comparison of the RT-qPCR and RNA-seq. N, adjacent non-tumor tissues. T, tumor tissues. Data are median ± SD. ^∗^*p* < 0.05, ^∗∗^*p* < 0.01, ^∗∗∗^*p* < 0.001, ^∗∗∗∗^*p* < 0.0001 are based on the Student’s *t*-test.

To validate the results of the RNA-seq, we selected 23 dysregulated RNA candidates including mRNAs, lncRNAs, miRNAs and circRNAs for RT-qPCR validation with 21 HCC patient sample pairs (Figure 3A–D). Housekeeping gene ACTB was used as the endogenous control. In the mRNA group, ZIC5, C12orf75, C1QL1, TMEM74, and GNAZ were significantly upregulated in tumor tissues compared to the adjacent control; PZP and FAM65C were significantly downregulated compared to the adjacent control (Figure 3A). In the lncRNA group, lncRNA-TOB2P1, LOC100499489, lnc-NMRAL2p, and lnc-NBPF22P were markedly upregulated in tumor tissues, while LINC01093, LOC100130899, LOC200772, and lnc-FENDRR were significantly downregulated in tumor tissues, compared to the adjacent controls (Figure 3B). In the miRNA group, miR-10-3p was significantly upregulated in tumor tissues, while miR-200a-3p, miR-200b-3p, and miR-139-5p were significantly downregulated in tumor tissues (Figure 3C). In the circRNA group, circAKR1B10 and circAKR1C3 were significantly upregulated in tumor tissues, while circHMGCS1 and circC3P1 were markedly downregulated, compared to the adjacent controls (Figure 3D). For these 23 RNAs examined with 21 pairs of patient samples, the RT-qPCR verification was in accordance with RNA-seq results with high confidence (Figure 3E).

**FIGURE 4:**
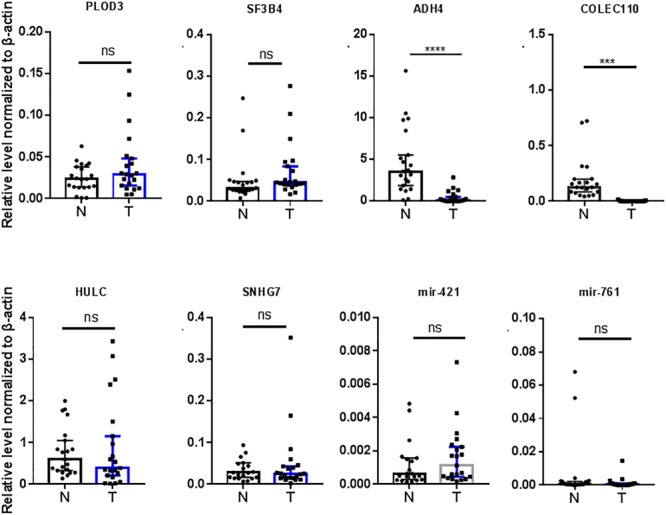
Validation of RNAs previously reported by other studies in 21 HCC tumor tissues by RT-qPCR. N, adjacent non-tumor tissues. T, tumor tissues. Data are median ± SD. ns, not significant, ^∗∗∗^*p* < 0.001, ^∗∗∗∗^*p* < 0.0001 are based on the Student’s *t*-test.

We also validated those transcripts with aberrant levels in HCC such as mRNAs previously identified by other studies (Figure 1C), with the same 21 HCC patient sample pairs with RT-qPCR (Figure 4). Among the eight transcripts (PLOD3, SF3B4, ADH4, COLEC10, HULC, SNHG7, miR-421, and miR-761), only two (ADH4 and COLEC10) transcripts were in accordance with RNA-seq results and previous reports (Figure 4; Wei et al., 2012; Zhang and Wu, 2018).

## Discussion

### Co-expression Network of lncRNAs/mRNAs and GO Analysis

**FIGURE 5:**
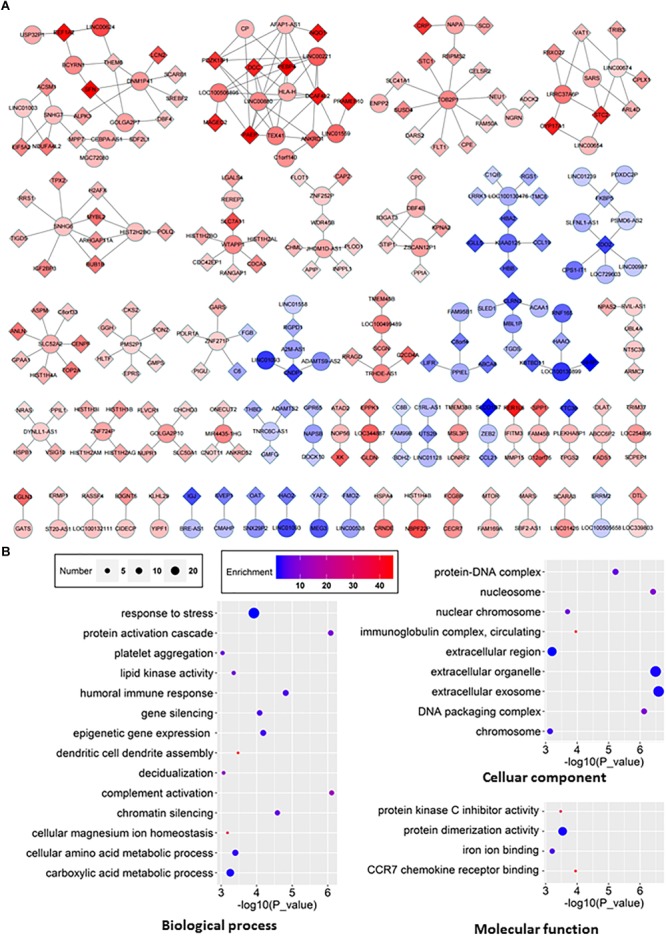
The lncRNA-mRNA network in HCC and GO analyses of dysregulated genes. **(A)** The co-expression network between differentially expressed lncRNA and mRNA (correlation coefficient absolute value ≥0.99). The boxes represent mRNA, the circles represent lncRNA. Red, upregulated; blue, downregulated. **(B)** GO analyses and annotation of dysregulated genes in three main categories: biological processes, cellular components and molecular functions.

We then set out to investigate the identified lncRNAs associated with HCC. LncRNA functions can be predicted based on the functions of their co-expressed protein-coding genes, and alterations in the associations between these genes in clinical samples can be used to identify key lncRNAs in HCC (Cabili et al., 2011; Liao et al., 2011; Guo et al., 2013). We constructed a co-expression network of lncRNAs and co-expressed mRNAs based on the RNA-seq data to investigate their interactions (Figure 5A). Our analysis demonstrated that 98 lncRNAs interacted with 175 mRNAs (Figure 5A). The results of GO pathway analyses of the differentially RNAs showed that most co-expressed lncRNAs were closely related to several important pathways, including biological processes such as gene silencing, chromatin silencing, and response to stress, cellular components such as extracellular region, and molecular functions such as protein dimerization activity (Figure 5B).

### Construction of CeRNA Network

**FIGURE 6:**
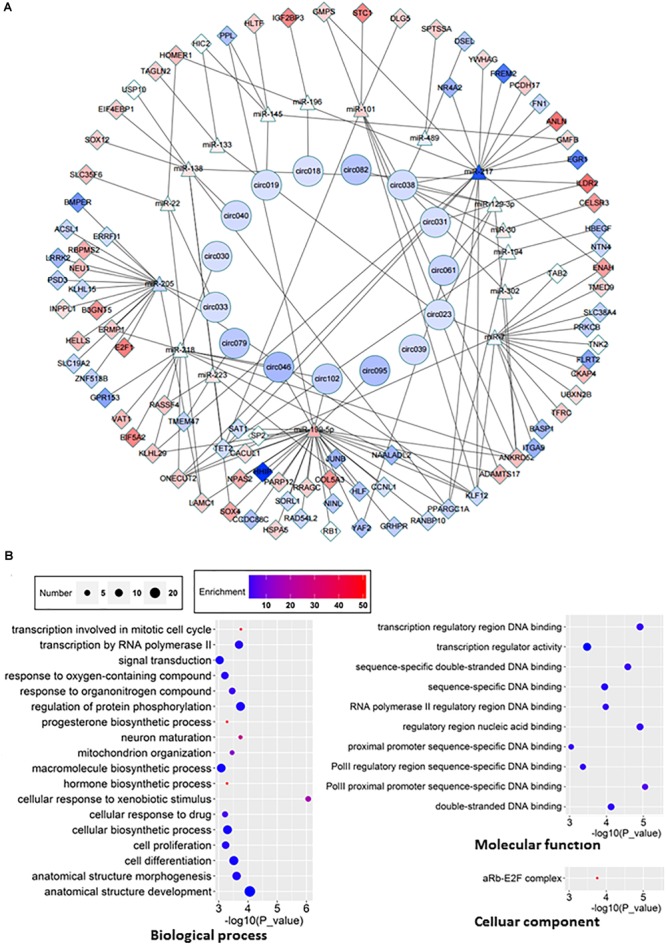
ceRNA network and GO analyses of related genes. **(A)** ceRNA network was constructed according to the interactions among circRNAs, mRNAs, and miRNAs. Boxes represent dysregulated mRNA, triangles represent dysregulated miRNA, circles represent dysregulated circRNAs. Red, upregulated; blue, down-regulated. **(B)** GO analyses of affected pathways in three main categories: cellular components, biological processes and molecular functions.

One of molecular functions of circRNAs is ceRNA (Hansen et al., 2013; Memczak et al., 2013; Piwecka et al., 2017). In order to investigate the potential circRNAs acting as ceRNAs in HCC through regulating miRNAs and consequently modulating mRNAs, we constructed a ceRNA network among differentially expressed circRNAs, miRNAs, and mRNAs in HCC (Figure 6A). 15 circRNAs (Table 2), 17 miRNAs, and 89 mRNAs were found to be correlated in this ceRNA network. The results of GO pathway analyses showed that mRNAs in this network were correlated to regulations of biological processes such as protein phosphorylation, signal transduction, and cell proliferation, molecular functions such as transcription regulator activity and double-stranded DNA binding, and cellular components such as Rb-E2F complex (Figure 6B).

**Table 2:** Information of circRNAs in Figure 5A.

circRNA	Genomic position	Gene symbol	circRNA ID
circ018	chr2 120885263 120932580	GLI2	Novel
circ019	chr13 33109905 33111164	N4BP2L2	hsa_circ_0100273
circ023	chr8 18725183 18730243	PSD3	hsa_circ_0136098
circ030	chr16 18852886 18856973	SMG1	hsa_circ_0006434
cir031	chr10 13322975 13337606	PHYH	hsa_circ_0093118
circ033	chr5 139819703 139828890	ANKHD1	hsa_circ_0001541
circ038	chr21 16386664 16415895	NRIP1	hsa_circ_0004771
circ039	chr12 12672795 12674397	DUSP16	hsa_circ_0003855
circ040	chr7 70227855 70236630	AUTS2	hsa_circ_0080420
circ046	chr6 161006077 161012133	LPA	hsa_circ_0131238
circ061	chr19 41382431 41414558	CYP2A7	Novel
circ079	chr5 78324255 78329231	DMGDH	hsa_circ_0129713
cir082	chr9 136302868 136303486	ADAMTS13	hsa_circ_0089372
circ095	chr6 161010584 161016567	LPA	Novel
circ0102	chr6 161006077 161016567	LPA	Novel

### Survival Curves of Identified Genes

**FIGURE 7:**
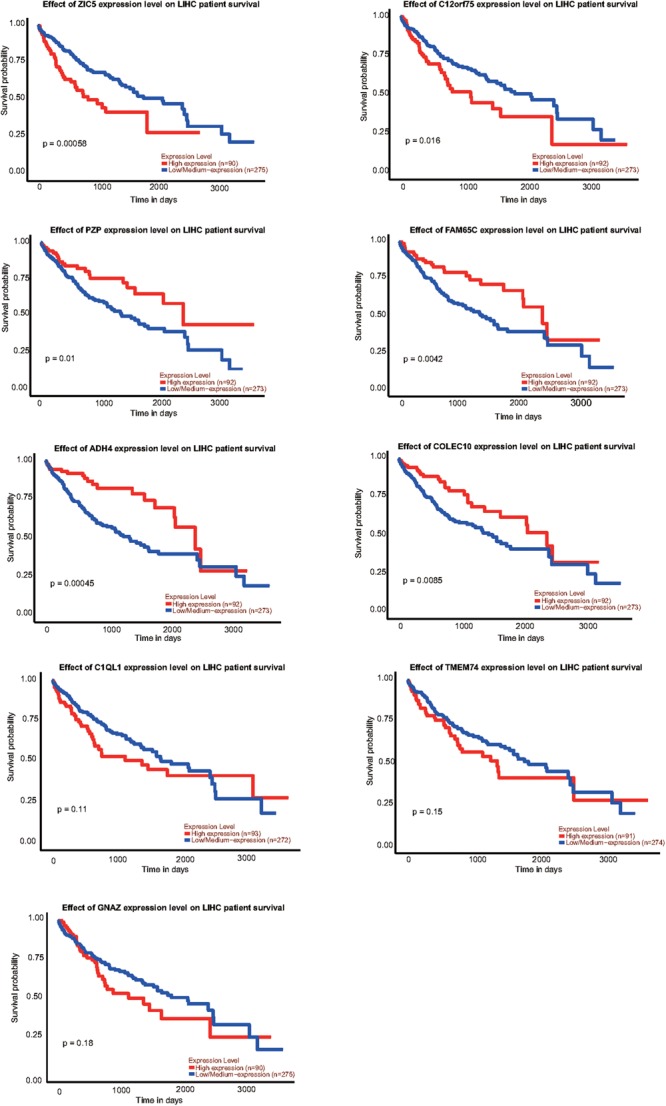
Survival curves of identified genes analyzed by UALCAN analysis; *p*-value was calculated by log-rank test.

To explore the relationship between our identified targets and clinical observations, we then examined with survival curves in online database, UALCAN analysis^2^. Total of 9 mRNAs, 7 of them identified in our RNA-seq and qRT-PCR verification and 2 of them previously identified and further verified by this study, were analyzed with survival curves. 6 (ZIC5, C12orf75, PZP, FAM65C, ADH4, and COLEC110) out of the 9 mRNAs were correlated to survival curves with significance in HCC (Figure 7). Those genes we found upregulated in HCC (ZIC5, C12orf75) were positively correlated with survival curves (patients with higher expression levels in HCC survive shorter). Genes downregulated in HCC (PZP, FAM65C, ADH4, and COLEC110) were negatively correlated with survival curves (patients with higher expression levels in HCC survive longer). For other identified genes including significantly upregulated/downregulated mRNAs, lncRNAs, miRNAs, and circular RNAs, the UALCAN database does not have HCC-relevant information about them.

The incidence of HCC has been increasing, and the consequent mortality is also rising for the past decades (Forner et al., 2018). For the early-stage patients, it is amenable to potentially curative treatments such as resection, liver transplantation, image-guided tumor ablation and systemic therapy, which can increase the 5 years survival rates to 60–70% (Llovet and Bruix, 2008; Yu, 2016). However, patients who are diagnosed at the advanced stage of HCC are treated poorly due to lack of effective treatment options for potential liver disease. Early diagnosis and effective surveillance are required for the treatment of HCC patients to reduce the disease-related mortality. Future diagnosis and treatments call for novel HCC biomarkers and potential targets.

RNA biomarkers, as measurable clinical indicators, can be used to predict and detect some diseases state and symptoms outside the body of patient with unique advances. To provide effective treatment for HCC patients and insights for future diagnosis, several potential RNA biomarkers for HCC have also been investigated (Klingenberg et al., 2017). Accumulating evidences have shown that lncRNA and miRNAs are suitable potential markers for HCC (Xie et al., 2016; Birgani et al., 2018). It has been reported that more than half of the miRNAs genes are located in cancer-associated genomic regions or in fragile sites (Greene et al., 2017). Aberrant expression of lncRNAs, miRNAs, and circRNAs along with mRNAs may directly or indirectly lead to the progression of cancers due to their massive involvement (Ryan et al., 2010; Cheetham et al., 2013; Guarnerio et al., 2016).

RNA-seq is a powerful tool to study and detect the global transcriptome in tissues and cells (Sharma et al., 2010; Adey et al., 2013). In this study, we have identified 919 differentially expressed mRNA, 207 lncRNAs, 216 miRNAs, and 152 circRNAs in HCC through RNA-seq, these dysregulated RNAs, especially those validated with 21 patient HCC samples can be highlighted as potential biomarkers or therapeutic targets for HCC (Figure 3).

Although increasing pieces of evidence have demonstrated the role of aberrant expression of mRNA, miRNA, lncRNA, and circRNA in HCC, not many studies have systematically investigated the crosstalk among transcripts in this context. The co-expression network between lncRNA and mRNA (Figure 5A) and the ceRNA network of differential expression circRNA-miRNA-mRNA (Figure 6A) in our study provide insights for further investigation. Of course, both networks have their own limitation. For example, circRNAs can function not only as ceRNAs but also as transcriptional regulators (Li Z. et al., 2015; Hu and Zhou, 2018). Another interesting point is that circRNAs seem to be more often downregulated in tumor tissues as shown in this study as well as several other studies (Figure 2D; Zheng et al., 2016; Greene et al., 2017).

We examined the 8 RNAs reported in other studies, however only two were found consistent with our RT-qPCR results from 21 patients (Figure 4). The inconsistence may be due to the fact that all patients in this study were HBsAg positive with advanced stage HCC, and our patient cohort may be distinct from previous studies. We did not have an opportunity to investigate the potential exposure of the main HCC carcinogen aflatoxin of these patients, which may be a weakness of this study. Another limitation of the present study is that, just like most Chinese HCC patients, all patients in this study are already in the advanced stage upon their first diagnosis, due to limited coverage of preclinic screening. We also explored the correlation of the RNAs identified in our study with patient survival curves. 6 out of the 9 mRNAs were correlated to survival curves of HCC, indicating multiple transcripts identified in this study may play critical roles in the tumorigenesis and advance of HCC (Figure 7).

## Conclusion

In conclusion, we have provided a comprehensive identification and analyses of the differentially expressed mRNAs, miRNAs, lncRNAs, and circRNAs using RNA-seq, and some of these transcripts have been verified with clinic HCC samples. Functional network of lncRNA-mRNA and circRNA-miRNA-mRNA ceRNA network have been systematically established to further indicate potential interactions in HCC. GO pathway analyses also facilitate future studies on the specific mechanisms of HCC. We expect this work will serve as a valuable resource in future clinical diagnosis and therapy of HCC.

## Ethics Statement

This study was carried out in accordance with the recommendations of the Human Research Ethics Committee of University of Science and Technology of China (USTCEC201700007) with written informed consent from all subjects. All subjects gave written informed consent in accordance with the Declaration of Helsinki. The protocol was approved by the Human Research Ethics Committee of University of Science and Technology of China (USTCEC201700007).

## Author Contributions

GX, GS, and LC designed and initiated this project. GS provided the major funding. GS and LC supervised the experiments. ZS and XW performed all the experiments. ZS, XW, LC, and GS analyzed the data and wrote the manuscript. All authors have discussed the results and made comments on the experiment.

## Conflict of Interest Statement

The authors declare that the research was conducted in the absence of any commercial or financial relationships that could be construed as a potential conflict of interest.

## Footnotes

1 www.cytoscape.org

2 http://ualcan.path.uab.edu/analysis.html
